# Reply to Letter to the editor: “Utilization of CT and MRI scanning in Taiwan, 2000–2017”

**DOI:** 10.1186/s13244-023-01478-7

**Published:** 2023-08-21

**Authors:** Fransisca Fortunata Effendi, Chung-Chien Huang, Wing P. Chan

**Affiliations:** 1 Department of Health Care Administration, College of Management, Taipei Medical University, Taipei, Taiwan; 2 Department of Medical Quality, Wan Fang Hospital, Taipei Medical University, Taipei, Taiwan; 3 Department of Radiology, Wan Fang Hospital, Taipei Medical University, Taipei, Taiwan; 4 Department of Radiology, School of Medicine, College of Medicine, Taipei Medical University, Taipei, Taiwan

**Keywords:** Computed tomography, Diagnostic imaging, Magnetic resonance imaging, Epidemiology, Utilization

Dear Editor in Chief,

We thank Li et al. [1] for their interest in our study [2]. We really appreciate the feedback and guidance for future work.

First, with regards to using the entire population of Taiwan as the denominator for calculating utilization rates, this is actually in accordance with OECD data, which also uses the entire population as the denominator in the formula to calculate utilization rates. Therefore, the comparison is apples-to-apples.

Accordingly, based on what has been conveyed in Table 1, the source of the data in Table 1 for Taiwan has been classified by hospital or medical urban center, this should not be misconstrued to depict the whole population of Taiwan. In addition, we would be unable to utilize the number of yearly users of ED services in Taiwan, as records only note CT usage and are not adjusted to the individual level.

Furthermore, because almost 99.8% of Taiwan’s population is covered under the NHI, the possibility of underestimation of imaging use due to self-pay and undercoding is unlikely to be nearly as significant in Taiwan as it is in the US.

Second, we do understand that including CT usage data that directly comes from the single-payer billing database would yield better results. This is one limitation that we have already admitted.

Third, as we have the limited access to other countries’ databases, understanding the complexities in comparing Taiwan’s CT and MRI utilization rates with those of other OECD countries is going to be limited. We are incorporating what we have available to provide insights on similar rates for the readers of our study.

## Data Availability

Not applicable.
